# Association of leptin and insulin resistance in PCOS: A case-controlled study

**Published:** 2017-07

**Authors:** Bahia Namavar Jahromi, Mohammad Hassan Dabaghmanesh, Mohammad Ebrahim Parsanezhad, Faranak Fatehpoor

**Affiliations:** 1 *Infertility Research Center, Shiraz University of Medical Sciences, Shiraz, Iran.*; 2 *Department of Obstetrics and Gynecology, School of Medicine, Shiraz University of Medical Sciences, Shiraz, Iran.*; 3 *Endocrine and Metabolism Research Center, Shiraz University of Medical Sciences, Shiraz, Iran.*; 4 *Department of Endocrinology, School of Medicine, Shiraz University of Medical Sciences, Shiraz, Iran.*; 5 *Student Research Committee, Shiraz University of Medical Sciences, Shiraz, Iran. *

**Keywords:** Leptin, Polycystic ovarian syndrome, Infertility, Insulin resistance

## Abstract

**Background:**

Endocrine abnormalities related to polycystic ovary Syndrome (PCOS) are important problems.

**Objective:**

To compare serum leptin levels between infertile women with and without PCOS. To rank sensitivity of six indirect methods for detection of insulin resistance (IR) and to evaluate the association between leptin and IR in PCOS group.

**Materials and methods:**

This Case-controlled study performed on 189 infertile women referred to Shiraz Mother and Child Hospital during 2012-2015. Ninety-nine PCOS cases according to Rotterdam criteria were compared to 90 cases without PCOS. Serum leptin, body mass index (BMI), several hormones, and their correlation coefficients with leptin were compared. IR in PCOS women was measured by indirect methods, including fasting blood sugar (FBS), fasting insulin (FI), glucose/insulin, homeostatic model assessment of insulin resistance (HOMA-IR), quantitative insulin sensitivity check index (QUICKI), and MacAuley index. Association between IR and leptin was evaluated. Independent sample t-test and Pearson’s test were used.

**Results:**

Infertile women with PCOS had higher BMI (26.47±3.62 vs. 24.82±5.18 kg/m^2^) and serum leptin levels (41.79±187.89 vs. 19.38±12.57 ng/mL). Leptin showed significant association with weight and BMI in both groups (p<0.001) and to age in non-PCOS group. HOMA-IR showed the highest rate of IR followed by FI and QUICKI methods. The mean leptin levels had positive association with IR assessed by HOMA-IR (p<0.001), QUICKI (p<0.001), FI (p=.002), and FBS (p=0.02).

**Conclusion:**

BMI and IR have positive association with serum leptin in PCOS infertile women. HOMA-IR followed by FI and QUICKI is the most sensitive test for detection of IR.

## Introduction

Polycystic ovary syndrome (PCOS) is the most common consequence of chronic anovulation with the prevalence of approximately 5-10% worldwide (1, 2). PCOS may have different etiologies and complex pathogeneses with various clinical presentations during the life span. PCOS is an important clinical topic in the fields of gynecology and reproduction (oligo/anovulation, hirsutism, infertility), metabolics (insulin resistance (IR), type II diabetes, cardiovascular risks), psychology (anxiety, depression), and nutrition and life style (obesity, decreased quality of life) (3, 4). 

The diagnostic criteria for PCOS were first suggested by the National Institutes of Health in 1990 and were revised by the 2003 Rotterdam Consensus Workshop (5). Presence of two of the following three criteria is needed for the diagnosis: 1- menstrual abnormalities (amenorrhea, oligomenorrhea), 2- clinical and/or biochemical hyperandrogenism, and/or 3- the ultrasound appearance of polycystic ovaries (6).

About 35-60% of PCOS women are obese. Indeed, central or android obesity is associated with IR, hyperinsulinemia, type II diabetes, hyperandrogenemia, metabolic syndrome, and infertility. Evaluation of glucose intolerance and IR are recommended for PCOS patients with premature adrenarche. Hyperinsulinemic euglycemic clamp as the gold standard method for evaluation of IR is difficult to be performed in clinical situations (7). Therefore, several other indirect methods are suggested for detection of IR, including fasting insulin (FI), fasting blood sugar (FBS)/FI, Homeostatic Model Assessment of Insulin Resistance (HOMA-IR), Quantitative Insulin Sensitivity Check Index (QUICKI), and MacAuley index (MCA) (8-11).

However, all these methods have their own limitations and there is no general agreement on the best and most practical method. Leptin, a peptide produced by fat cells, acts on the brain and controls metabolic rate, appetite, and food intake. Leptin plays a major role in the normal physiology of the reproductive system (12-14). Circulating leptin concentration is regulated by insulin (15).

This study aims to compare infertile women with and without PCOS regarding serum levels of leptin and several other hormones. The association between serum leptin levels and patients’ age, BMI, and other hormone levels are also evaluated. Besides, the rate of detection of IR in the infertile women with PCOS was ranked by six different methods to find the most sensitive one.

## Materials and methods

This case-controlled study was conducted on 189 infertile women referred to the Infertility Clinic at Shiraz Mother and Child Hospital affiliated to Shiraz University of Medical Sciences from May 2012 to August 2015. The participants were selected by simple random sampling. 

Ninety-nine infertile women who fulfilled the criteria of PCOS using Rotterdam criteria were enrolled into the case group, while 90 cases without the criteria of PCOS were allocated to the control group (5, 6). All PCOS women in this study showed at least 10-12 antral follicles in each ovary by transvaginal ultrasound scan accompanied by at least one of the two other signs; i.e., oligomenorrhea or amenorrhea and/or clinical or biochemical signs of hyperandrogenism. A women was included only if she had stopped metformin or any other medication at least two months before blood collection.

Five mL of blood from antecubital vein of every participant was taken and the sera were extracted and frozen. Serum leptin levels were measured by ELISA test (by using BioVendor ELISA kit, Germany). Additionally, leutinizing hormone (LH), follicle stimulating hormone (FSH), thyroid stimulating hormone (TSH), and prolactin levels were measured by immune radiometric assay (RIA kit IRMA tube, Korea). Testosterone levels were also measured by radioimmunoassay (Testo-RIA kit, France) and the values were compared between the study groups.

FI, FBS, and lipid profiles were measured only for the PCOS group and a descriptive analysis was performed. Insulin level was measured by immunoradometric assay (Insulin IRMA kit, Hungary). Moreover, six indirect methods were employed for assessment of insulin resistance in the PCOS patients, including FBS, fasting insulin, glucose/insulin ratio, HOMA-IR, QUICKI, and MacAuley tests. The formula used for calculation of HOMA-IR was: HOMA= fasting insulin (µ U/mL)×fasting glucose (mg/dL)/405, which was considered abnormal when was more than 2. 

The QUICKI value is also the inverse of the sum of the fasting glucose and fasting insulin concentrations expressed logarithmically as: QUICKI= 1/ (log insulin+log glucose in mg/dL), which was considered abnormal when ≤0.33. However, MacAuley was based on the increase of TG and fasting insulin calculated as: McAuley (McA)= exp [2.63-0.28 ln (insulin in mU/L)-0.31 ln (triglycerides in mmol/L)], with the abnormal values considered to be ≤5.8 (8). FBS≥100, FI≥10 and glucose/Insulin<4.5 were considered as cut of points in favor of IR. After all, the most sensitive method was selected and sensitivity, specificity, positive predictive value (PPV), and negative predictive value (NPV) were computed for other tests compared to it.

**Ethical consideration**

This study was approved by the Ethics Committee (EC-P-91-4059) and Institutional Review Board of Shiraz University of Medical Sciences. Complete explanations were provided and written consent forms were obtained.

**Statistical analysis**

All statistical analyses were performed using the SPSS software (Statistical Package for the Social Sciences, version 22.0, SPSS Inc, Chicago, Illinois, USA) and p<0.05 was considered to be statistically significant. Considering α= 0.05, β= 0.2, and power= 80% and using the following formula, the sample size was set at 98 participants for each of the case and control groups.

$n=\frac{{\left(Z_{\frac{\alpha }{2}}+Z_{1-\beta }\right)}^{2}(S_{1}^{2}+S_{2}^{2})}{{\left(\mu_{1}-\mu_{2}\right)}^{2}}$

Independent sample t-test was used to compare the two groups regarding demographic and hormonal characteristics and leptin values and to compare the association between leptin level and IR shown by six indirect methods in the PCOS patients. Besides, Pearson’s test was used to assess the correlation between leptin level and other parameters in the PCOS and non-PCOS groups. Furthermore, sensitivity, specificity, PPV, and NPV of the tests were calculated using Receiver operating characteristic curve analysis and were compared to HOMA-IR as the most sensitive test.

## Results

This study was performed on 189 infertile women divided into two groups; 99 women with and 90 women without the criteria of PCOS. The two groups were compared with respect to age, BMI, duration of infertility, and serum hormonal levels. The means of BMI (p= 0.034), serum leptin levels (p= 0.032) and FSH levels (p= 0.019) were significantly higher in the PCOS group, while, other parameters had no statistical difference (Table I).

In the PCOS group, 9 out of the 99 women were obese with BMI ≥30 (9.09%) and 31 (31.3%) were overweight with 25≤ BMI ≤29.9. In the non-PCOS group, on the other hand, 11 out of the 90 women (12.2%) had BMI ≥30 and 28 (31.1%) had 25≤ BMI ≤29.9. This implies that the frequency of obese or overweight women was not higher in the PCOS group in our study, but the overall mean BMI values were higher among the PCOS group participants.

To find any possible correlations between serum leptin levels and other parameters, correlation coefficients were calculated. Leptin levels were significantly correlated to the women’s weight and BMI (p<0.001) in both PCOS and non-PCOS groups. Serum leptin level was also positively correlated to the women’s age in the non-PCOS group (p=0.01). The mean values for FBS, FI, Glucose/ Insulin, HOMA-IR, QUICKI and MacAuley Index were 99.90±11.18, 11.52±6.13, 12.08±10.60, 2.87±1.65, 0.33±.03 and 6.93±2.29 in the PCOS group, respectively. The number and frequencies of IR in the PCOS group that were calculated by the six methods showed that HOMA-IR >2 was the most sensitive method and included 62 (65.5%) women table II. Also, sensitivity, specificity, PPV, and NPV of the tests were calculated using Receiver operating characteristic curve analysis and were compared to HOMA-IR, as the most sensitive test (Table II). 

To find the association between leptin and IR the number of the cases diagnosed to have IR by each method was assessed Table III. The mean leptin levels were calculated in each group and compared between the IR and non-IR cases. The mean leptin levels were significantly higher among the patients with IR. HOMA-IR showed to be the most sensitive method by detecting 62 cases while glucose/insulin detected only three cases to have IR.

**Table I:** Comparison of the PCOS and non-PCOS infertile women regarding demographic and hormonal characteristics and leptin values

	**PCOS infertile women**	**Non-PCOS infertile women**	**p-value**
Age (year)	27.64 ± 4.34	29.35 ± 5.95	0.05
BMI (kg/m^2^)	26.47 ± 3.62	24.82 ± 5.18	0.034
Duration of infertility (year)	6.62 ± 3.89	6.45 ± 5.59	0.83
LH(mIU/mL)	7.74 ± 7.94	8.66 ± 21.62	0.56
FSH (mIU/mL)	5.63 ± 2.57	11.70 ± 44.52	0.019
TSH (mIU/L)	2.56 ± 2.06	2.55 ± 2.61	0.95
Prolactin (ng/mL)	24.20 ± 52.72	27.05 ± 68.72	0.66
Testosterone (ng/dL)	1.18 ± 2.99	2.14 ± 9.86	0.22
Leptin (ng/mL)	41.79 ± 187.89	19.38 ± 12.57	0.032

The values are presented as mean ± SD. Independent sample t-test was used for the analysis.

BMI: body mass index

LH: luteinizing hormone

FSH: follicle stimulating hormone

TSH: thyroid stimulating hormone

PCOS: polycystic ovary Syndrome

**Table II:** The number and percentage of insulin resistant patients in the PCOS group using indirect measurement methods also sensitivity, specificity, PPV, and NPV of other tests compared to HOMA-IR are calculated

	**FBS >100**	**Fasting insulin >10**	**Glucose/Insulin <4.5**	**QUICKI ≤0.33**	**McAuley** **≤5.8**	**HOMA-IR >2**
Insulin resistant	33 (34.7%)	52 (52.5%)	3 (3.2%)	49 (51.6%)	28 (28.3%)	62 (65.3%)
Non-insulin resistant	62 (65.3%)	47 (47.5%)	92 (96.8%)	46 (48.4%)	71 (71.7%)	33 (34.7%)
Total	95 (100%)	99 (100%)	95 (100%)	95 (100%)	99 (100%)	95 (100%)
Sensitivity	45.2%	80.6%	4.8%	79.0%	37.3	-
Specificity	84.8%	100.0%	100.0%	100.0%	93.9	-
PPV	84.8%	100.0%	100.0%	100.0%	92.0	-
NPV	45.2%	73.3%	35.9%	71.7%	44.3	-

The values are presented as N (%).

FBS: Fasting blood sugar

PCOS: Polycystic Ovary Syndrome

PPV: Positive Predictive Value

NPV: Negative Predictive Value

QUICKI: Quantitative Insulin Sensitivity Check Index

HOMA-IR: Homeostatic Model Assessment of Insulin Resistance

**Table III:** The association between leptin level and insulin resistance (IR) calculated by six indirect methods in the PCOS patients

**Methods**	**Number of the patients with IR**	**Serum leptin level in ng/mL**	**p-value**
FBS≥100 as IR	33	25.52 ± 9.96	0.182
FBS<100	62	20.23 ± 9.60	
Fasting insulin≥10 as IR	52	24.95 ± 9.38	0.002
Fasting Insulin <10	47	18.57 ± 9.78	
HOMA-IR≥2 as IR	62	25.42 ± 9.63	<0.001
HOMA-IR<2	33	16.01 ± 7.62	
Glucose/insulin<4.5 as IR	3	29.91 ± 15.19	0.269
Glucose/insulin≥4.5	92	21.95 ± 9.91	
QUICKI≤3.8 as IR	49	25.50 ± 9.17	<0.001
QUICKI>3.8	46	18.61 ± 9.73	
McAuley≤5.8 as IR	28	25.18 ± 8.62	0.069
McAuley>5.8	71	20.74 ± 10.30	

Values are presented as: Mean±SD. Independent sample t-test was used for statistical analyses.

IR: Insulin Resistance

FBS: Fasting Blood Sugar

HOMA-IR: Homeostatic Model Assessment of Insulin Resistance

QUICKI: Quantitative Insulin Sensitivity Check Index

## Discussion

Fat cells secrete leptin as a major endocrine organ. Leptin was discovered to regulate weight first in 1950. Ob-gene responsible for obesity in rats synonymous to LEP gene in human was discovered in 1994 (16, 17). About 60% of PCOS women are obese and obesity is associated with hyperandrogenism, IR, and metabolic syndrome. In our study, PCOS women had higher means of BMI and leptin levels compared to the controls (18-20).

Our results revealed a significantly lower mean level of FSH in the PCOS patients compared to the controls that supports the previous studies. However, the mean prolactin levels had no significant difference between the two groups as previously reported (20). Generally, LH and testosterone levels are usually higher in PCOS women (21, 22). Nonetheless, our study showed no significant difference between the two groups for the mean levels of LH and testosterone (Table I). Also, obesity defined as BMI≥30 was detected in 9.09% of the PCOS women compared to 12.3% of the non-PCOS ones. These findings might be due to the fact that our PCOS patients were highly motivated infertile women who had tried different healthy diet and lifestyles or hormonal managements before referral to our infertility center.

Our results indicated a significant association between leptin levels and BMI in both PCOS and control groups, supporting the previous studies (22). Logically, increase in fat cells and BMI is accompanied by increase in leptin secretion. This study demonstrated no significant association between leptin and testosterone levels as previously reported (22, 23). Dyslipidemia and obesity are common metabolic abnormalities in PCOS women (19, 24). So the first generally accepted step for treatment of infertility with PCOS is weight reduction.

In the present study, six indirect methods were used for measurement of IR for every PCOS woman. Confirming our results previous studies showed that FBS was not a good indicator for IR. Some studies have also shown that FBS concentration was not different between PCOS and control groups (20). Prior investigations showed higher levels of fasting insulin and HOMA-IR in PCOS cases compared to the controls (19, 21, 22). The overall prevalence of IR in PCOS has been reported to be between 50-75% in the previous studies. In our study, FBS >100 was detected in only 34.7% of the PCOS women in favor of its low sensitivity confirming a previous study (7). In addition, glucose/ insulin ratio and MacAuley index has lower sensitivities. Considering the fact that improving lifestyle and weight reduction play important roles in prevention and management of PCOS consequences, we decided to choose HOMA-IR with the most sensitivity as was suggested before (25). We compared other methods to HOMA-IR and found that fasting insulin and QUICKI tests had the highest sensitivities and specificities following HOMA-IR (Table II). Glucose/insulin ratio had the lowest sensitivity (4.8%), as reported by a previous research (26).

In the recent years, the cut-off values for FBS have been reduced from 140 to 126 in order to increase the sensitivity of the test for detection of diabetic patients. By adjustment of the cut-off values of the aforementioned tests, more sensitivity will be achieved for better detection of IR. Although our subjects had been on diet and tried for weight reduction before the study, IR was still highly detected in favor of impaired glucose metabolism in PCOS.

The mean leptin levels were significantly higher among the patients with IR (Table III). There are few studies that reported the correlation between leptin and IR in PCOS (25, 27). We believe that more fat cells produce higher serum leptin levels associated with higher IR in PCOS women with high BMI. We had only a few missing values that still may be considered to be a limitation for this study. Also, we admit that if we had a larger sample size, the results would be more precise. Moreover, the lipid profiles, FBS, and insulin levels were only measured for the PCOS group and, consequently, they could not be compared to the non-PCOS women. We suggest further studies to confirm these results. Findings of this study shows positive correlation between IR, BMI and serum leptin levels. We suggest HOMA-IR to be checked in every PCOS woman followed by lifestyle changes for IR cases. 

## Conclusion

BMI and IR have positive association with serum leptin in PCOS infertile women. HOMA-IR followed by FI and QUICKI is the most sensitive test for detection of IR.

## Note

***This article extracted from M.D. thesis. (Faranak Fatehpoor)***
